# Mast cell in infantile hemangioma

**DOI:** 10.3389/fonc.2024.1304478

**Published:** 2024-01-19

**Authors:** Meng Xia, Wenying Liu, Fang Hou

**Affiliations:** Department of Pediatric Surgery, Sichuan Provincial People’s Hospital, School of Medicine, University of Electronic Science and Technology of China, Chengdu, China

**Keywords:** infantile hemangioma, mast cell, estradiol, proangiogenesis, anti-angiogenesis

## Abstract

Infantile hemangioma (IH) is the most common benign vascular tumor characterized by three phases — proliferation, early involution and late involution. Mast cells (MCs) play an important role in allergic reactions and numerous diseases, including tumors. While the mechanisms underlying MCs migration, activation and function in the life cycle of IH remain unclear, previous studies suggested that MCs circulate through the vasculature and migrate into IH, and subsequently mature and get activated. Estradiol (E2) emerges as a potential attractant for MC migration into IH and their subsequent activation. In various stages of IH, activated MCs secrete both proangiogenic and anti-angiogenic modulators, absorbed by various cells adjacent to them. Imbalances in these modulators may contribute to IH proliferation and involution.

## 1 Introduction

Infantile hemangioma (IH) is the most common benign vascular tumor among infants, impacting nearly 10% of all infants worldwide (1, 2). IH is more prevalent in females, premature births, Caucasian descent, and low birth weight infants (1, 2). Despite its prevalence, the exact cause of IH remains elusive. IH comprises stem cells capable of proliferating and differentiating into various cell types, including neuroglial stem cells, endothelial progenitor cells, hematopoietic stem cells, and mesenchymal stem cells (3). IH exhibits three overlapping phases: proliferation (proliferating) phase, early involution (involuting) phase, and late involution (involuting) phase (4). Histologically, the proliferation phase is characterized by a lot of rapidly dividing endothelial cells (ECs), plump pericytes, mast cells (MCs), and interstitial dendritic cells. The early involution phase exhibits increased MCs, apoptotic bodies, and decreased mitotic figures, along with a reduction in lesional capillaries and ECs. As involution continues into the late phase, ECs, MCs, and vascular channels gradually diminish, replaced by loose fibrous or fibrofatty tissues (4). This spontaneous regression is the most important biological feature of IH. Although approximately 90% of IH cases resolve spontaneously, about 69% are left with varying degrees of sequelae, such as hyperpigmentation, telangiectasia, fibrofatty tissue accumulation, and scarring (5).

MCs, recognized for their involvement in allergic reactions and various physiological processes, including angiogenesis, vasodilation, wound healing, fibrosis, and immune regulation, have also garnered attention in the context of diseases, including tumors (6). Over a century ago, Ehrlich and Westphal reported the presence of MCs in human tumors for the first time (7). Extensive evidence suggests that MCs accumulating around tumors can either promote or inhibit tumor growth, contingent on local stromal conditions and the stage of tumorigenesis (8). However, their association with IH remains somewhat contentious. Some researchers have reported that MCs promote the proliferation of ECs in IH by secreting multiple modulators (9), while others have contended that MCs inhibit IH ECs proliferation through the release of anti-angiogenic factors (10). In this review, we delve into recent findings regarding MCs origin, migration, activation, and reactivity within the context of IH.

## 2 Origin and localization of mast cells

MCs were initially thought to originate from blood basophils or local histiocytic progenitors (11). Paul Ehrlich made the pioneering discovery of MCs and documented their presence in perivascular tissues in 1877 (12). Lombardo’s study in 1909 revealed that MCs first appear in human embryos during the eighth week of gestation (13).

Subsequently, various cells, including ECs, mesenchymal cells, thymocytes, pericytes, and fibroblasts, have been identified as precursors to MCs (14, 15). Unlike T and B lymphocytes, monocytes, and neutrophils that circulate in the bloodstream, MCs are tissue-resident guard cells (16). They are distributed throughout nearly all tissues, with a particularly prominent presence in the dermis, respiratory mucosa, digestive and urogenital systems, blood and lymph vessels, and fibroblasts. This distribution allows them to detect changes in their local microenvironment (17). The presence of MCs within vessel walls underscores their role in the vasculature, notably in vasodilation and tissue-specific responses to circulating agents (18).

## 3 Mast cells migrate into IH

As previously mentioned, MCs originate from progenitors in the bone marrow and enter the peripheral blood, but they are not yet fully mature at this stage. Circulating MC precursors migrate into various vascularized peripheral tissues via adhesion contacts to a network formed by integrins (19). Subsequently, they take up residence within these tissues, completing their maturation and function under the influence of locally produced factors (20, 21). Studies have indicated that the number of MCs fluctuates during the different pathological phases of IH (22, 23). Indeed, the count of MCs is highest during the early involuting phase, followed by the late involuting and proliferating phases, respectively (24). Furthermore, the percentage of proliferating MCs is highest in the proliferating phase and lowest in the late involuting phase (23). Nevertheless, the precise mechanisms underlying the presence and fluctuations in MC numbers within IH remain unclear.

A recent observation regarding the presence of Estradiol (E2) on MCs suggests that E2 might play a role in attracting MCs to migrate into IH tissue (24). This is likely because MCs primarily migrate to their target locations in response to locally produced chemokines (21, 25, 26), and E2 could potentially modulate the expression of chemokine receptors on the surface of MCs (20). Upon migrating into IH tissue, MCs undergo maturation and activation. A previous study reported an abundance of immature MCs during the IH proliferation phase, while the involution phase sees a rich infiltration of mature MCs (4).

## 4 Activated MCs in IH

MCs may be activated through various mechanisms, leading to the secretion of a wide array of mediators, including proteases (such as tryptase, chymase, carboxypeptidase, MCP-1, MCP-2), proteoglycans (like heparin and chondroitin sulfate), proteins (including CRH, osteopontin, thymic stromal lymphopoietin), biogenic amines (histamine and serotonin), growth factors (PDGF, VEGF, FGF2, bFGF, NGF, PAF), and cytokines (such as TNFα, lymphotactin, IL-1β, IL-3, IL-4, IL-5, IL-6, IL-9, IL-10, IL-12, IL-13, IL-16, IL-17A, IL-18, IL-23, IFN-α, IFN-β, IFNγ) (6, 20, 25, 27).

**Figure 1:**
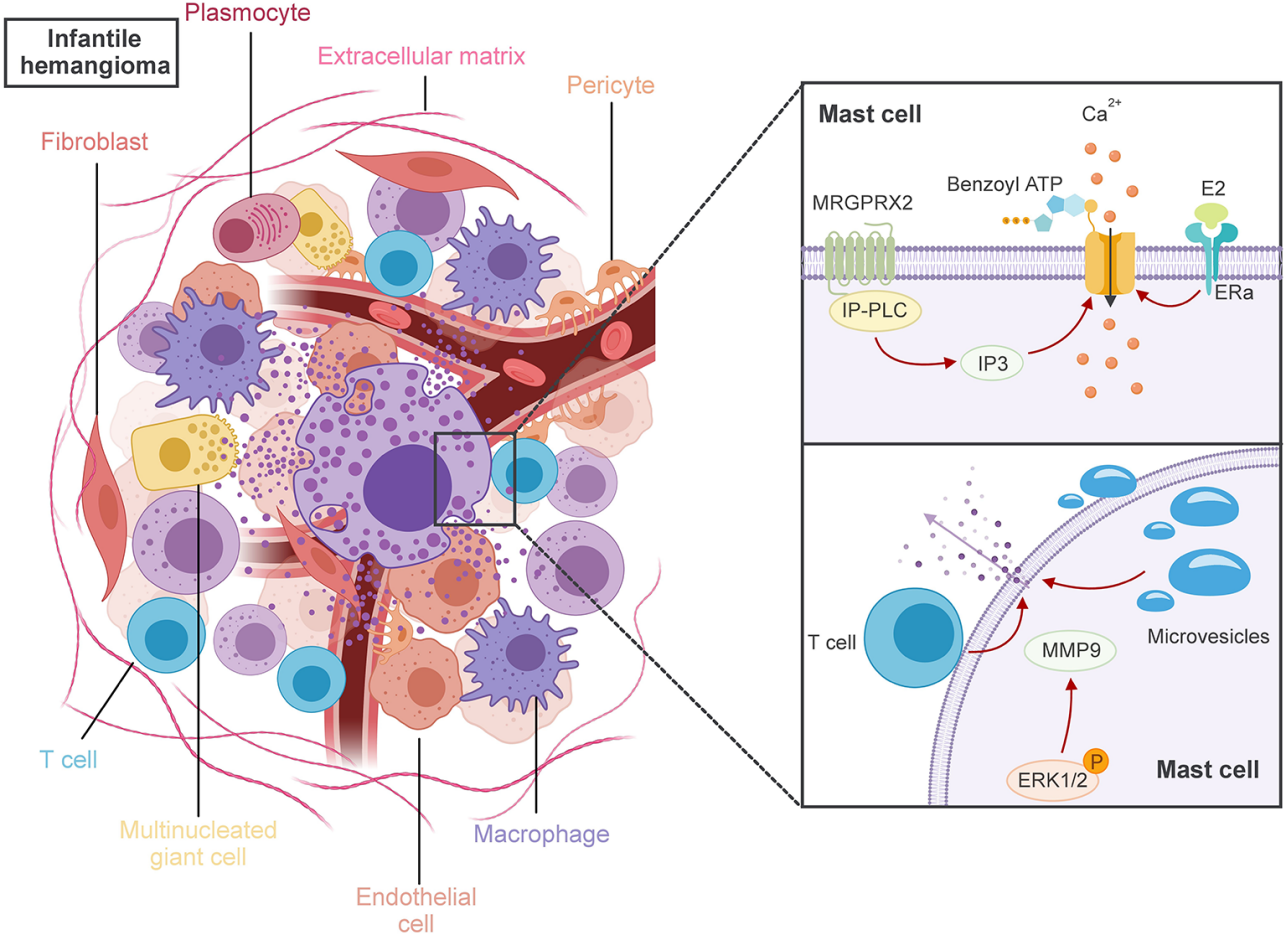
MCs have crucial roles to play in the pathogenesis of IH. MRGPRX2 binding triggers IP-PLC and leads to the formation of IP3. The binding of benzoyl ATP activates ion channels, and E2 binding to ER-a on MCs, all of which could induce the inflow of extracellular Ca2+, ultimately leading to the activation of MCs degranulation. Physical contact with activated T-cell membranes and released microvesicles triggers the degranulation of MCs, which in turn stimulates the activation of ERK1/2. These actions ultimately lead MCs to release either angiogenesis or anti-angiogenesis key factors during various IH phases, which are taken up by adjacent cells like fibroblasts, macrophages, multinucleated giant cells, and plasma cells.

MCs degranulation is essential for the release of these mediators. This complex process involves membrane fusion and various proteins, depending on whether allergic or non-allergic reactions trigger it (28). Recent advances have elucidated three primary pathways of degranulation activation in non-allergic reactions (27) (**Figure 1**):

1. Activation through Mas-related G-protein-coupled-ligand receptor member X2 (MRGPRX2) binding, which triggers Inositol Phosphate-Phospholipase C (IP-PLC) and leads to the formation of Inositol trisphosphate (IP3). This results in the opening of Ca^2+^ channels, elevating intracellular Ca^2+^ levels and initiating degranulation.
2. Activation by binding of Benzoyl ATP activates ion channels, causing a rapid influx of nonselective ions, including Ca^2+^, and the opening of large plasma membrane pores, ultimately resulting in degranulation.
3. Activation of Extracellular signal-regulated kinase 1/2 (ERK1/2) due to physical contact with activated T-cell membranes and released microvesicles, leading to the expression of cytokines, chemokines, adenosine, and growth factors.

Despite these insights, the precise mechanisms behind MCs activation and the number of activated MCs in IH remain elusive. Some literature suggests that local elevated levels of E2 may influence MCs activation (29). It has been reported that E2 binding to estrogen receptor-a (ERa) on MCs may trigger a rapid onset and progressive influx of extracellular Ca^2+^, leading to MCs activation (30). In this context, a previous report suggests that E2-positive MCs might be involved in the population of activated MCs in IH (24).

Upon activation, MCs can release a diverse group of pivotal factors that play crucial roles in either angiogenesis or anti-angiogenesis during the various phases of IH (31). Those factors will be uptake by the adjacent cells, such as fibroblasts, macrophages, multinucleated giant cells, and plasma cells within IH (32). However, the specific angiogenesis or anti-angiogenesis factors exchanged between these cells remain unclear. As a general rule of thumb, MCs release mediators based on the ligands that activate their receptors and the surrounding microenvironment (18).

## 5 Mast cells are involved in IH proliferation

The notion that MCs may promote tumor growth has long been suggested (33) and has been observed to induce the proliferation of human microvascular ECs (34). Activated MCs release multiple modulators, including histamine, tryptase, chymase, type VIII collagen, vascular endothelial growth factor (VEGF), and fibroblast growth factor (FGF)-2, which have been implicated as angiogenic factors in IH (9).

Type VIII collagen and FGF-2 are believed to play significant roles in IH ECs proliferation, with their expression detected on MCs only during the proliferation phase of IH. These findings align with recent *in vitro* research showing that propranolol can inhibit IH proliferation by reducing the expression of VEGF-A, bFGF, MMP2, MMP9, and tryptase in MCs (35).

There are three types of specific proteases present in MCs secretory granules: chymase, tryptase and carboxypeptidase A (31). Chymase indirectly induces angiogenesis by activating MMP-9 or converting Angiotensin I to Angiotensin II. Tryptase serves as a significant factor in angiogenesis. It can alter the composition of the extracellular matrix by stimulating its components, leading to neovascularization (36). In IH, the percentage of MCs positive for both tryptase and chymase reaches highest during the proliferation phase, and decreases in early and late involution phases, indicating a proangiogenic role for chymase in IH (36). Histamine, a vasoactive amine synthesized and stored in MCs’ cytoplasmic granules, contributes to vasodilation, arteriolar constriction, angiogenesis, and vascular permeability (18). Despite the above knowledge, confirming the proangiogenic role of tryptase and histamine in IH remains a great challenge. MCs express histamine and tryptase throughout all phases of IH (31). The total number of MCs positive for tryptase peaks during the involution phase of IH (24, 31).

In addition to secreting mediators upon activation, human skin MCs can spontaneously and constitutively release pro-angiogenic factors (37). Meanwhile, IH arise most commonly in or under the skin.

## 6 Mast cells are involved in IH involution

Activated MCs also release multiple anti-angiogenic modulators, including interferon (IFN)-α, IFN-β, IFN-γ, and transforming growth factor (TGF)-β. FGF-2 and VEGF are recognized as significant angiogenic factors in proliferating IH. IFN-α and IFN-β can down-regulate FGF-2 (38, 39), while IFN-γ inhibits the mitogenic effect of VEGF (40). TGF-β can inhibit ECs proliferation, and a lack of TGF-β may lead to increased ECs proliferation (10, 31). In IH, the expression of TGF-β in the early involution phase was significantly higher than the late involution and proliferation phases (41). Furthermore, TGF-β1 can stimulate the proliferation of fibroblasts (42), As fibrosis occurs in the IH involution phase, cytoplasmic bridges between MCs and fibroblasts have been observed by ultrastructural studies (31).

Moreover, the number of MCs positive for clusterin/apolipoprotein J (an apoptotic protein) is highest during the early involuting phase and absent in the proliferating phase of IH (23). Recent studies have observed that IH in the involuting phases contains more E2-positive MCs compared to proliferating phase specimens (24). These findings suggest that certain MCs, activated by E2, probably play a role in IH regression.

Propranolol, a non-specific beta-blocker, has gained recognition as the first-line treatment for IH, resulting in rapid lesion regression and reduced fibrofatty residuum. While the molecular mechanisms underlying propranolol’s action on IH are not fully understood, some research suggests that MCs, through secreted proteases, may contribute to accelerated microvascular maturation, enhanced tissue remodeling, and reduced fibrofatty residuum observed with propranolol treatment (43). Prey S et al. (44) studied the expression of beta-adrenergic receptors (ADRB1, 2 and 3) to identify potential propranolol targets cells. They found that ADRB2 was highly and uniformly expressed on MCs of IH. MCs, due to their high ADRB2 expression, may be potential targets for propranolol and may play an indirect anti-angiogenic role in IH (45).

Furthermore, the significant increase in MCs numbers following steroid treatment of IH suggests that MCs may possess antiangiogenic properties and expedite IH involution (46), although propranolol’s proven efficacy and safety in treating IH have reduced the use of steroids (47).

## 7 Conclusions

Throughout the years, researchers have been captivated by the biology and the role of MCs in IH. Several significant discoveries have shed light on the function of this unique cell in IH. Nevertheless, numerous questions remain unanswered. E2, a female sex hormone, has long been suggested to influence IH and MCs functionality (24, 33). Moreover, IH, like many MC-related diseases, exhibits a higher prevalence in females than in males (26, 48). However, the well-documented connections among E2, MCs, and IH continue to elude us. Recent robust data indicate that imbalanced levels of angiogenic and anti-angiogenic factors secreted by activated MCs may contribute to IH proliferation and involution. Therefore, the coming years hold the potential for rapid advancements in MCs research, offering insights not only into their relationship with E2 but also a deeper understanding of their role in IH.

## Author contributions

MX: Writing – original draft. WL: Writing – review & editing. FH: Writing – review & editing.
